# Researchers’ and Clinicians’ Perceptions of Recruiting Participants to Clinical Research: A Thematic Meta-Synthesis

**DOI:** 10.14740/jocmr1619w

**Published:** 2014-03-31

**Authors:** Lisa Newington, Alison Metcalfe

**Affiliations:** a NIHR Biomedical Research Centre, Guy’s and St Thomas’ NHS Foundation Trust and King’s College London, Guy’s Hospital, SE1 9RT, UK; b Florence Nightingale School of Nursing and Midwifery, King’s College London, James Clerk Maxwell Building, Waterloo Road, SE1 8WA, UK

**Keywords:** Research subject recruitment, Research personnel, Medical staff, Systematic review

## Abstract

**Background:**

Recruiting the desired number of research participants is frequently problematic with resulting financial and clinical implications. The views of individuals responsible for participant recruitment have not been previously reviewed. This systematic review and thematic meta-synthesis explores researchers’ and clinicians’ experiences and perceptions of recruiting participants to clinical research, with the aim of informing improved recruitment systems and strategies.

**Methods:**

Studies published between January 1995 and May 2013 were identified from: Ovid MEDLINE, Ovid EMBASE, Ovid PSYCHINFO, ASSIA, British Nursing Index, Scopus, Web of Science, CINAHL and PubMed. Included studies were original peer reviewed research, with qualitative methodologies and an aim of exploring the views of clinicians and/or researchers on recruitment to clinical research. Studies discussing the recruitment of patients unable to give informed consent were excluded. The findings sections of the relevant studies were free coded to identify key concepts which were grouped into hierarchical themes. The quality of the identified studies was assessed and the relative contribution of each paper was checked to ensure individual studies did not dominate in any theme.

**Results:**

Eighteen relevant papers were identified which examined the views of researchers and clinicians in 10 clinical specialties. Five main themes emerged: building a research community, securing resources, the nature of research, professional identities and recruitment strategies. The views of researchers and clinicians were similar, although the role of ‘researcher’ was inconsistently defined.

**Conclusions:**

The general experience of recruiting participants to clinical research was one of competition and compromise. Competition arose over funding, staffing and participants, and between clinical and research responsibilities. Compromise was needed to create study designs that were acceptable to patients, clinicians and researchers. Forging relationships between clinical and research teams featured extensively, however the involvement of patients and the public within the research community was rarely discussed.

## Introduction

The concept of evidence-based practice in healthcare necessitates the completion of robust research in answer to important clinical questions. However, not all studies are able to recruit a sufficient number of participants to adequately answer their research question in the allocated time-period, raising issues of resource usage and delaying changes to practice. Only 31% of a cohort of UK-based multicentre trials conducted between 1994 and 2002 achieved their original recruitment target [1], and this situation is not unique to the country, study design or clinical area [2, 3].

A recent systematic review and meta-analysis of strategies to improve recruitment to randomized controlled trials (RCTs) found 45 studies exploring this issue [4]. The majority of studies involved strategies aimed at potential trial participants, such as comparisons of different information delivery formats or consent processes. The remaining four studies assessed the effect of greater contact between the central trial team and the recruiting sites [5, 6] or the use of recruitment training packages [7, 8], but none of these interventions were associated with significant increases in the number of participants recruited.

The reasons why some individuals consent to participate in clinical research whilst others decline are rapidly being explored. Proposed factors include age [9], ethnicity [10], availability of healthcare [11] and health beliefs [12]. Interviews with research participants have shown different levels of research awareness prior to taking part, identified mixed experiences of participation and raised varied suggestions of ways to improve recruitment [13]. Increasing participation in clinical research is recognized as a key agenda within the NHS to facilitate evidence-based policy, improve health outcomes and reduce inequality [14]. The ‘OK to ask’ [15] and ‘get randomized’ [16] campaigns have been launched to promote public awareness of research and encourage patients to discuss research opportunities with their clinicians.

For the clinicians responsible for recruiting patients to clinical research, additional workloads, financial losses, conflicts of interest and lack of knowledge about the individual study have all been discussed as barriers to successful research [17, 18]. Conversely, financial incentives, positive attitudes towards research, relevance to their patients and effective information technology systems have been proposed as facilitators [19]. To date, the views of research teams working in different clinical specialities, and on studies of different designs, have not been systematically reviewed and synthesized, with much of the existing literature focusing on RCTs [4, 20]. It is hoped that the identification and synthesis of experiences across different study designs will enable better understanding of the issues involved in participant recruitment and inform improvements in general recruitment systems and strategies. This review therefore posed the following question: what are researchers’ and clinicians’ experiences and perceptions of recruiting participants for clinical research?

## Methods

Empirical studies exploring researchers’ and/or clinicians’ views of recruitment to clinical research were systematically identified and the findings from relevant papers were combined in a thematic synthesis based on the process outlined by Thomas and Harden [21]. Several survey-based studies have been conducted to determine the views of clinicians on various aspects of clinical research, for examples see King et al [22], Meropol et al [23] and Ford et al [24], however it was chosen to limit the current review to qualitative research to allow a less structured and more in-depth exploration of the phenomena involved. Where studies adopted a mixed methods approach, only the findings from the qualitative aspect were included in this review.

### Search strategy

**Figure 1:**
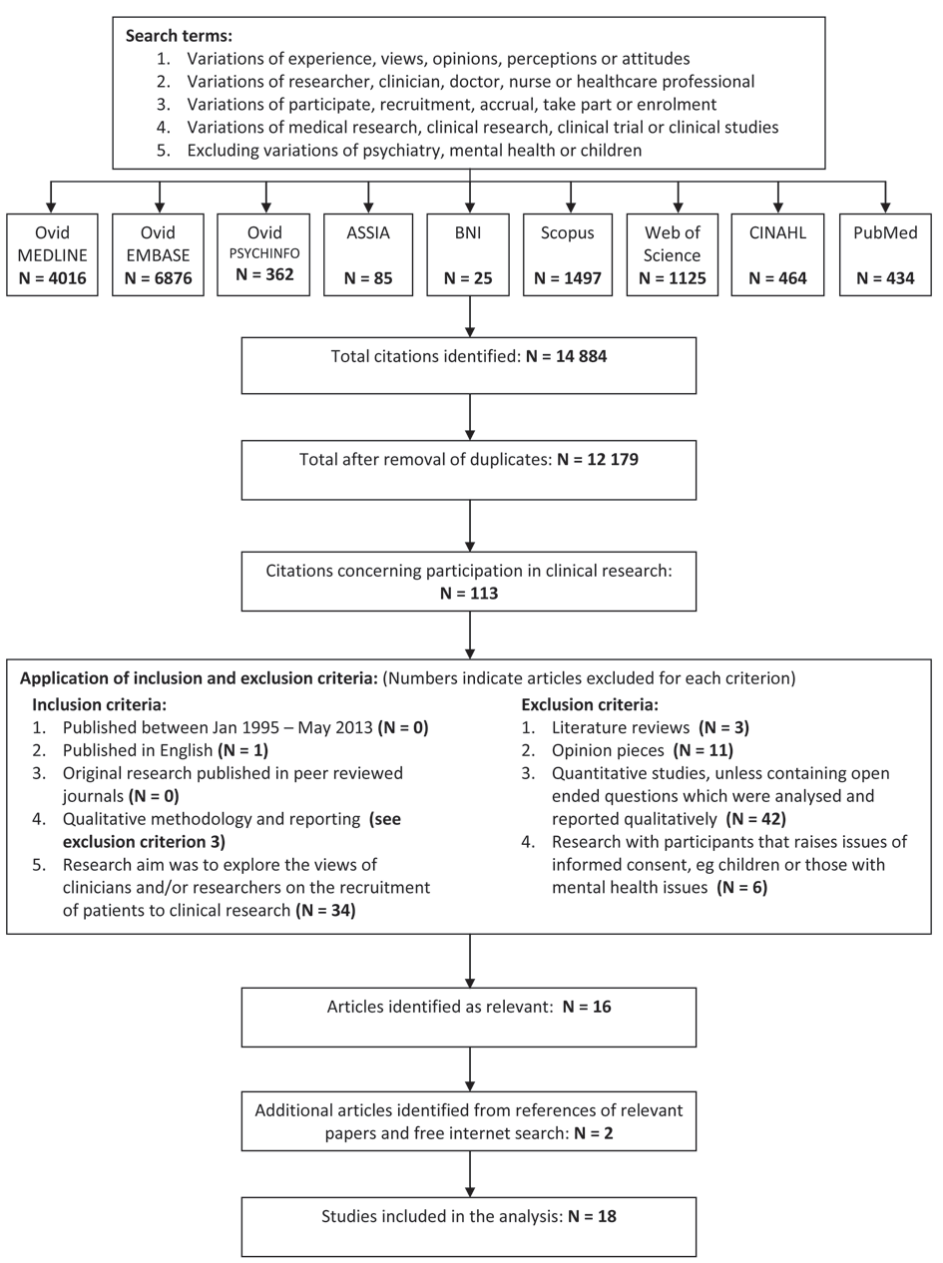
Search strategy and identification of studies included in the review.

Relevant articles published between January 1995 and May 2013 were identified by the primary author using electronic databases, hand-searching the references of eligible papers and free internet searches [25]. Search terms are listed in Figure 1, with the full strategy shown in Supplementary 1 (www.jocmr.org). This process was duplicated and reviewed by the second author. The lower boundary of 1995 was chosen because substantial changes to the NHS research structure were introduced after the 1994 Culyer report [26]. Changes to USA research processes also arose during this period following lobbying from patient groups concerned about research accessibility and regulatory delays [27]. This changing context is also likely to apply to other jurisdictions and it was felt that earlier research may not adequately reflect the current situation. Figure 1 shows a flowchart of the identification and selection process including the inclusion and exclusion criteria and the numbers eliminated at each stage. Relevant articles were original peer reviewed studies reporting qualitative research that explored the views of researchers and/or clinicians on the recruitment of participants for clinical research. Clinical research was defined as any medical research requiring consent to participate, including the donation of tissue samples, observational studies and clinical trials. Studies discussing the recruitment of individuals who may not have capacity to give informed consent, such as children, intensive care patients or those with mental health issues, were excluded due to the additional ethical issues surrounding the recruitment of these populations [28]. The included papers were limited to English language.

### Assessment of quality

The assessment of quality in qualitative research has received much debate and agreement on the most suitable method has not been reached [29]. To allow the systematic identification of study weakness, methodological flaws and missing information, eligible articles for this review were assessed for quality using the CASP checklist [30]. The aim was not to exclude studies on grounds of quality, rather to allow an assessment of the impact of study quality on the review’s findings [21]. After the thematic synthesis was complete, the relative contribution of the included studies was assessed to ensure that the key aspects of the analytic themes were not derived from a small number of papers, or papers of poorer quality.

### Analysis

All relevant articles were imported into NVivo (QRS, version 10) and the qualitative findings for each were free coded line-by-line to identify the key concepts. The similarities, differences and links between the resulting codes were used to form groups of codes and create a hierarchical structure representing distinct themes [21]. Consistency was checked at each stage by reviewing the codes against the original text and re-coding earlier codes where appropriate. The initial findings were organized according to the identified themes and discussed by both authors prior to the development of the final version.

## Results

A total of 18 studies were included in the review [31-48], the details are outlined in Supplementary 2 (www.jocmr.org). Oncology was the most frequently mentioned clinical specialty [32, 34, 35, 38, 41, 43, 45, 48] and clinical trials were the most common type of research discussed [32, 34, 35, 37, 38, 41, 43-46, 48]. The term ‘researcher’ was not clearly defined, with some studies combining the views of recruiting doctors and nurses, principal investigators and trial managers [34], others looking solely at the perspectives of research assistants or associates [33, 38, 48], and others exploring the experience of research nurses [35, 44, 47] or scientists [40]. Similarly, the studies focused on a range of clinicians, including pharmacists [36], doctors [31, 32, 37, 41, 43, 44], midwives [39], nurses [46] and the whole multi-disciplinary team [45]. For this reason, it was not possible to differentiate the views of researchers and clinicians throughout, but where possible this has been discussed.

Researchers’ and clinicians’ experiences and perceptions of recruiting participants for clinical research centered around five main themes: building a research community, securing resources, the nature of research, professional identities and recruitment strategies.

### Building a research community

It was widely recognized that successful clinical research could not be a unilateral activity. Both researchers and clinicians acknowledged the need to engage all relevant parties (researchers, clinicians, patients and public) in discussions regarding research aims and practices [31, 32, 34, 39, 40, 42, 43, 45, 47] and overall there was a common belief that clinical research was necessary to generate improvements in patient care. However, despite this shared goal, it appeared that the idea of collaborative research communities was more theoretical than established. For some clinicians, there appeared to be a disconnect between the belief that clinical research was required and the desire to be personally involved. Reasons included a perceived lack of resources [31, 34, 38, 43, 46], issues with the study question or design [38, 43], concerns that patients would ask questions outside the clinician’s knowledge base [36, 39] and the need to prioritize patient care [37, 39]. To counter this potential lack of interest, researchers saw it as their role to engage clinicians in each piece of research, to persuade them of its relevance and to encourage recruitment [34, 37, 40, 42, 47].

The benefit of being involved in a research community was felt by both professional groups. Where the clinicians had been actively engaged in the research decision-making the studies appeared well supported by clinicians [34, 40, 47]. The reverse was also true [43]. The formation of collaborative ties between researchers and clinicians was seen to create a sense of loyalty to the project, which in turn aided recruitment [34]; previous alliances and positive experiences were also viewed as beneficial [34, 37, 45, 46]. Less attention was given to establishing a collaborative relationship with the local population or target patient groups. Researchers from studies involving minority groups highlighted the value of pre-research trust-building activities, such as free screening sessions, holding meetings in the local community and involving key community members in research design and recruitment [35, 37].

The flow of information from the main study team to clinicians and participants was also viewed as part of the collaborative process. Keeping the recruiting clinicians informed of the research project was seen to facilitate recruitment by maintaining interest in the study [32, 46] and by boosting competition between recruiting sites [44]. Providing insufficient information to clinicians was viewed negatively and regarded as a lack of appreciation of their contribution [39]. Furthermore, both professional groups emphasized the importance of feeding back research findings to all parties and ensuring the results were used to improve patient care [31, 32, 39].

### Securing resources

Sufficient and appropriate resources were seen as an integral part of a successful research community. Money, staff, time and patients were all viewed as scarce commodities and strategies to secure them were generally seen as beneficial to recruitment. For researchers, there was a double aspect to recruitment: recruiting the appropriate healthcare teams or clinicians, who could then recruit eligible patients. In order to gain the support of clinical teams, it was essential that research collaboration did not impose additional financial costs or increase workloads [34, 43]. Providing a sufficient number of well-trained staff was therefore seen as central to the successful running of clinical research projects [42, 45].

Both researchers and clinicians recounted the limited scope of public funding, and obtaining financial support from commercial companies was seen as an important resource [34, 44]. Commercial funding was believed to facilitate patient participation by enabling compensation for all research costs, such as parking and childcare [38] and to aid the development of dedicated research teams through the creation of salaried research positions and the purchase of equipment or training not available through healthcare budgets [44]. However, researchers and clinicians were wary of the potential loss of control associated with commercially driven interests and the appeal of commercial funding was tempered by the need to maintain scientific and ethical integrity [34, 39, 44].

Eligible patients were also viewed as a scarce resource, creating competition between academic and commercially funded research for the recruitment of specific patient populations [41]. Competition between centers was mentioned as a driving force for recruitment [44], although professional rivalry was seen to prevent cooperation thereby reducing overall recruitment [43].

### Nature of the research

The focus of the research was identified as a key factor in determining the recruiters’ contribution to a particular study [32, 34, 36-38, 40, 41, 43-45, 48]. Building collaborative research communities may aid the development of research questions that are truly relevant to clinicians and their patients; however, as discussed previously, it was important not to allow commercial agendas to dominate [34]. A conflict also arose between the use of a scientifically desirable study design and the creation of a study that was acceptable to recruiters and potential participants [34, 37, 38, 40]. There was agreement that RCTs were the gold standard for clinical effectiveness research, although several clinicians expressed concern about randomly allocating treatment, especially in the absence of clinical equipoise [31, 32, 43, 45]. There were fewer issues with the research design for other methods [39, 40, 42], possibly because clinicians had control over treatment decision making in these cases.

In addition to valuing the research question and accepting the study design, researchers and clinicians must also be prepared to follow the study protocol. Clinicians from one study admitted deviating from the protocol in an attempt to prevent attrition and achieve good outcomes, despite understanding the impact this had on the robustness of the research and extrapolation of the findings [44]. These clinicians were driven by competition with other recruiting centers, the desire to gain future commercial funding and the belief that certain patients would benefit from additional treatment [44].

Suggestions for more user-friendly research for both clinicians and participants included adopting flexible study designs, such as those that allow placebo and control groups to have access to the intervention at a later stage [34], early discussion of the exclusion criteria to avoid patient disappointment after expression of interest [33], providing study interventions in-house, rather than sending patients to an external clinic [37], combining different study methods and approaches [42], creating a smooth and easy system for trial recruitment [38], having realistic recruitment targets [43] and avoiding intensive research protocols and tight time schedules [38].

Research regulations were seen as a necessary requirement to ensure good and ethical research practice, although it was hinted by both professional groups that these regulations were becoming excessive and a balance needed to be struck between protecting research participants and hindering beneficial research [38, 40-43]. The importance of informed consent and voluntariness were stressed by all studies and there was awareness that contextual aspects, such as the way a recruiter informs a patient and the situation in which recruitment occurs are influential to the potential participant’s decision [31, 32, 38, 39, 45, 48]. Problems were reported by clinicians who needed to inform patients and obtain their consent in a short timeframe [39], research assistants who felt that patients were too overloaded with information [33, 38] and multi-disciplinary teams who identified variations in the approach and effectiveness of ‘selling’ the trial to eligible patients [45].

### Professional identities

The distinction between researcher and clinician was not clear-cut, with clinical consultants acting as principal investigators and research nurses also having clinical responsibilities. These interlinking roles and responsibilities were seen as a dual identity of healthcare provider and researcher [34, 37]. Some individuals found this situation difficult to reconcile and remained suspicious of research, viewing it as an additional burden and a competitor for their patients’ attention [34, 36, 37, 43]. However, the majority welcomed the opportunity to be involved in the progression of clinical practice and also saw participation in research as providing patient benefit [34, 35, 43-48]. Researchers and clinicians viewed research involvement as an opportunity for personal career progression or to improve institutional status [40, 41, 44], however, some researchers commented on the lack of career structure and difficulty retaining experienced staff [42].

For healthcare professionals, the responsibility of being a patient advocate was central in their decision to approach a patient about research participation [32, 34, 37]. Where clinicians also held a principal investigator role, some reported a desire to delegate recruitment and retention activities to those with less of a stake in the project, such as research nurses, to minimize their conflicting responsibilities [44]. There were blurred boundaries between patient advocate and gatekeeping [42], with all recruiters reporting an element of gatekeeping for practical reasons, or because of their own implicit beliefs about the best interests of the patient, or in some cases, the best interests of the research. Reasons for limiting the inclusivity of recruitment included: concerns about the length of time it would take to explain the research because of language or psycho-social issues [38], an over emphasis on the vulnerability of the patient [42], concerns about gaining informed consent due to the educational level of the patient [43], perceived differences between eligibility criteria and patient suitability [41, 43, 46], cherry-picking patients to increase the chance of good outcomes [44], avoiding patients thought to be unreliable [44], deliberately choosing patients perceived as less compliant [46], avoiding patients already receiving a lot of care [46], fear of being regarded as coercive [45], the loss of personal equipoise [32], a general lack of enthusiasm about the research [48] and simply forgetting to ask [36, 39]. It was only a minority view that all patients are, and should be, free to make their own decision about research participation [32, 46].

Across the included studies, the responsibility for inviting patients to participate rested predominantly with the clinicians. It was emphasized that those involved in recruitment should have appropriate knowledge, including details of the research project and relevant regulations [33, 34, 40, 41]. There were instances where the recruiting clinicians felt they had too little or superficial information [39], while in other settings they felt more adequately prepared [46, 47]. It was argued for one study that researchers would be more appropriate recruiters given their knowledge of the aims and intricacies of the research [39, 40]. However, where research assistants were responsible for recruitment, they reported feeling ill-equipped to answer the patients’ medical questions and would have preferred clinician involvement [33].

### Recruitment strategies

Clinicians and researchers held similar views of the reasons why patients accept the invitation to participate in clinical research. Altruism was believed to be the main motivator, with the desire to help future generations [31, 32, 38, 42, 47] and the wish to give something back to their healthcare team [42] cited as potential explanations. Other proposed reasons included financial reimbursement [33, 38], the belief that participation will be personally beneficial, such as access to regular monitoring, access to new treatments or the chance of a cure [38, 45], or being in a situation with limited treatment options [48].

Suggested explanations of why patients decline to participate in clinical research were similar across both professional groups. Reasons included: a distrust of research and concerns about being a guinea pig [37, 38], disliking the idea of receiving a placebo [43, 48], having strong preferences for a particular treatment [43, 45] and issues with the additional time required for research participation, especially for patients with family or work commitments [38, 42, 47] or those living further away from the clinic [45]. In addition, one study found that eligible patients were reluctant to participate if they did not personally define themselves as suffering from the medical condition under investigation, whilst others felt that younger patients were more fearful and took longer to come to terms with their diagnosis making them harder to recruit [34].

The patient’s doctor [38, 47, 48], their family [38, 47] and the recruiting clinician [38, 40, 43, 45, 48] were all believed to make positive or negative contributions to recruitment, depending on their personal attitudes towards clinical research and their opinions of the particular study in question. Similarly, the effect of the media was variable depending on the nature of the coverage and general perceptions of the trustworthiness of the source [31, 37, 38, 48].

Numerous recruitment practices and strategies were discussed in the included studies. These strategies were broadly divided into three categories: emphasizing the benefits of research participation, engendering trust in the research and establishing effective systems for the recruiters, as shown in Table 1.

**Table 1:** Recruitment Strategies Identified

Recruitment strategy	Specific techniques
Emphasizing the potential benefits	Training recruiters to focus on study highlights [33]
	Neglecting to remind patients of randomization [32]
	Advertising participation as the only way to access a particular treatment [34]
	Focusing on elements important to each individual patient [47]
	Exploring what could be offered in return for participation [42]
	Offering flexible appointment dates and times [47]
Engendering patient support	Appealing to altruism [45, 47]
	Mentioning the study at an early stage of treatment [32, 44, 45]
	Providing positive messages about clinical equipoise and the importance of randomization [32, 45]
	Discussing the research in an unhurried manner in a stress free environment [32, 34, 39, 48]
	Recruiting in the community, rather than a clinical environment [37]
	Having dedicated and knowledgeable recruiters [45]
	Adapting recruitment strategies for individual patients [33, 34, 41, 47, 48]
	Being culturally sensitive and inclusive [37, 40, 42]
	Involving influential family or community members [47, 48]
	Visibly advertising the study and providing information in an accessible way [45, 48]
	Including stories from previous participants in the recruitment information [46, 47]
	Participating in strategies to increase public awareness of clinical research [45]
Establishing effective systems for recruiters	Providing regular research updates and reminders [34-36, 43, 46]
	Offering incentives for good recruitment [34, 35, 44]
	Allowing additional time for recruitment [46]
	Ensuring researchers, not clinicians, are responsible for labor intensive aspects of recruitment, such as trawling records and databases [34]
	Providing appropriate training [34, 36]

### Assessment of quality

The quality appraisal of the included studies is available in Supplementary 3 (www.jocmr.org). All themes were a composite of the findings of several studies and no one study was found to dominate. Studies of lower quality tended to have fewer participants and provided insufficient information about the data collection and analysis process.

## Discussion

This review has synthesized the perceptions and experiences of researchers and clinicians involved in recruiting participants to clinical research across a range of study designs, locations and clinical specialties. Despite these contextual differences, five common themes were identified: building a research community, securing resources, the nature of research, professional identities and recruitment strategies. While there were some differences in the issues highlighted by those working on different study designs, for example concerns about randomization and equipoise were only mentioned by those working on RCTs, none of the themes were dominated by reference to a particular study design. The current review also indentified an overall consensus between the perceptions and experiences of clinicians and researchers, which was unexpected given previous reports of the difficulty engaging clinicians in research recruitment [18, 49]. This finding may in part be explained by the overlapping roles of healthcare researcher and clinician, an issue which has also been recognized previously [50]. Alternative methods of reporting the opinions of those involved in recruiting participants for clinical research may be more informative, such as making a distinction between those who usually see the patient for routine clinical care and those only involved in the research study. However, this distinction was not easily identified across the studies included in the current review. One activity that was more commonly associated with researchers was the initial task of recruiting clinical teams to participate in their work. This was intricately linked to participant recruitment through the accepted influence of the clinician-patient relationship in a patient’s decision to take part, plus the more practical need to ensure that eligible patients were actually invited [51]. A recent systematic review of strategies aimed at improving clinician recruitment activities found the most promising interventions were aimed at increasing clinician research knowledge and engaging them in the recruitment process [20]. This mirrors the findings of the current review, where the idea of building a research community was seen as facilitating the continued interest of clinical teams thereby improving their participation in the whole research process.

It was evident that there were multiple instances where eligible patients were denied the opportunity to make their own decision about participating in clinical research. This did not solely take the form of clinician gatekeeping, which has been previously identified as problematic [52], but there were numerous instances where additional criteria were also applied by researchers to make a judgment on a patient’s suitability for their research. While this introduces the possibility of selection bias and reduces the generalizability of the findings [53], it is also inequitable. In England, the NHS Constitution pledges to “inform you of research studies in which you may be eligible to participate” [54]. From the studies included in this review, it appears that this may not always be the case and that more should be done to ensure potentially eligible patients are given the opportunity to make their own decision about research participation. The previously mentioned ‘Ok to ask’ and ‘get randomized’ campaigns are examples of this strategy and monitoring their effectiveness may aid the development of similar approaches elsewhere [15, 16].

There were no universally adopted recruitment strategies. This mirrors the findings of Treweek et al [4], who identified a range of techniques across studies specifically investigating optimal recruitment methods. Within the current review, the majorities of recruitment techniques were classified as attempts to engender patient support and were predominantly targeted at the individual patient level. Researchers and clinicians working with minority groups did highlight the importance of engaging their whole population, as have authors elsewhere [10, 55], but this concept was not discussed more widely. Traditionally, members of minority groups have been considered more difficult to recruit to clinical research, but throughout the studies reviewed all patients were viewed as a scarce resource. It is therefore surprising that more attention was not given to building a collaborative research community of clinicians, researchers and patients.

Altruism was suggested as the key principle explaining why patients decide to participate in clinical research. The significance of altruism and research participation has been discussed elsewhere and it is proposed that altruism is rarely the main motivational factor, with self-interest, encouragement from family and friends, and a sense of obligation to the study team also important [12, 56-58]. As the current review explored the opinions of recruiters, rather than research participants, it may be that the premise of altruistic research participation is a more idealistic than accurate interpretation of patients’ reasoning. However, research participants may also present a more idealistic view when questioned about their motivation for taking part [59]. The suggested dominance of altruistic motives contrasts with a key recruitment strategy also indentified in the current review: emphasizing the benefits of research participation. This inconsistency hints at disconnect between the recruiter’s theoretical view of research participation and the day-to-day practicalities of actually recruiting participants, a finding that warrants further investigation. The explanations given for patients declining to participate were a combination of logistical factors, such as a lack of time, in addition to more overarching concerns about the research process. This is consistent with other findings [51]. Greater involvement of patients and the public in research design and planning could help allay concerns about the processes of clinical research and thereby improve participation, but time will always be a barrier. The provision of financial compensation for the time involved in research participation may help offset this issue [4, 60, 61].

### Strengths and limitations

This review adds a new perspective by identifying and synthesizing the views of researchers and clinicians responsible for recruiting participants to research in different clinical areas, using different study designs. The implications for clinical research practice include the recommendation to ensure sufficient patient and public involvement within the research community, and the need to further explore more user-friendly research practices for both recruiters and participants.

The included studies contained the views of an array of clinicians and researchers; however the distinction between the two was less clear-cut than expected. Further work to investigate the recruitment practices and experiences of those usually involved in patient care and those solely working on a research project may shed more light on the perceived differences between patient advocates and gatekeepers.

The current review only included qualitative material. Several authors have published survey data in this field [22-24] and incorporating these findings in a mixed-methods review may provide additional themes. Furthermore, the included studies were limited to English language. The removal of this restriction may have enabled the identification of studies from a broader range of geographical locations; however preliminary work suggested a lack of relevant publications in alternative languages.

### Conclusions

The general experience of recruiting participants to clinical research was one of competition and compromise. Competition took the form of attracting sufficient funding, staff and participants, in addition to competing clinical and research responsibilities. Compromise was needed to create study designs that were acceptable to all involved, and to allow cooperation between commercial and academic institutions. Forging relationships between clinical and research teams featured extensively, however patient and public involvement in the research community was rarely discussed. Despite differences in location and healthcare systems, there were similarities between the reported recruitment experiences in all included studies. Whilst this hints at a common picture, given the majority of studies were located in Europe or North America, further work is required to assess the situation elsewhere.
